# Does Information Asymmetry Impact Sub-Regions’ Cooperation of Regional Water Resource Allocation?

**DOI:** 10.3390/ijerph16214292

**Published:** 2019-11-05

**Authors:** Li Pan, Xudong Chen, Lu Zhao, Anran Xiao

**Affiliations:** 1 Business School, Sichuan University, Chengdu 610064, China; panli@stu.scu.edu.cn (L.P.); 2018225020002@stu.scu.edu.cn (L.Z.); 2 College of Management Science, Chengdu University of Technology, Chengdu 610059, China; xiaoanran@cdut.edu.cn

**Keywords:** water allocation, bi-level optimization, regional cooperation, information asymmetry

## Abstract

Water resources allocation is an urgent problem for basin authorities. In order to obtain greater economic benefits from limited water supplies, sub-regions must cooperate with each other. To study the influence of cooperation among sub-regions and the symmetry of cooperation information on the interests of the basin authority and each sub-region, this study proposes a regional water allocation model in three different situations: (1) non-cooperation; (2) cooperation and information symmetry; (3) cooperation and information asymmetry. The proposed model clearly reflects the Stackelberg game relationship between the basin authority and sub-regions. Finally, the model is applied to the Qujiang River Basin in China, and the decisions of the basin authority and sub-regional managers of the Qujiang River Basin under three different situations are discussed. The results show that regional cooperation benefits both the cooperative regions and the social welfare value of the entire river basin, when compared with non-cooperation.

## 1. Introduction

Water is one of the most important resources for human survival, socio-economic development, and environmental sustainability (Bakker 2012 [1]). In recent years, with rapid development of the economy and society, population growth, and the development of urbanization, the demand for water resources has increased dramatically (Liu et al. 2016 [2]). By 2050, demand for water is projected to grow by more than 40% (Eliasson 2015 [3]). Due to the impact of climate change and increasingly frequent human activities, many regions of the world are facing serious water shortages. The imbalance between water supply and demand has become a major constraint to economic development of these regions and has even resulted in disputes among water users. Therefore, rational allocation of water resources is an important means to mitigate the water crisis. Basin water allocation should consider the principles of fairness, sustainability, and efficiency to achieve a sustainable and safe society (Slaibi and Silverbrand, 2009 [4]; Roa-Garcia and Maria, 2014 [5]; Xu et al., 2013 [6]).

In recent years, in order to improve the efficiency of water resources allocation and alleviate the imbalance between supply and demand of water resources, many scholars have proposed a multi-objective optimization model for basin water resource allocation based on equity, economics, and sustainability (Liu et al., 2010 [7]; Hu et al., 2016 [8]; Li and Guo, 2014 [9]). However, in practical watershed management systems, basin authorities and multiple sub-regional managers are often involved. As a leader, the basin authority is responsible for the sustainable development of the entire basin and needs to design a reasonable initial water rights allocation plan. Based on these initial water rights, sub-regional managers along the same basin act as followers to maximize the economic benefits of the areas they manage. Therefore, the basin authority can influence the decisions of sub-regional managers by adjusting the initial water rights, while the decision-making of water managers of each sub-area also affects the achievement of the larger goals. This interactive decision fits within the leader-follower framework of a Stackelberg game (Stackelberg, 1934 [10]), and can be represented by a bi-level programming model (Wen and Hsu, 1991 [11]). In order to reflect the Stackelberg game relationship between the upper and lower layers of water resources distribution in the basin, some researchers began to widely use the bi-level programming model (Table 1). For example, Xu and co-workers, 2017 [12] presented a bi-level optimization waste load allocation programming model under a fuzzy random environment to assist integrated river pollution control. Hu and co-workers, 2016 [13] developed a multi-objective bi-level model with the upper level reflecting the river basin authority’s allocation principle of equity and stability and the lower level aimed at ensuring maximal economic benefit efficiency for each sub-area.

As the demand for water continues to increase, the pressure on sub-regions to gain more water rights from river basin managers has also increased. When developing water withdrawal plans for water users, sub-regional water managers often consider how to allocate limited water resources to water users to obtain greater economic benefits. The economic benefits generated by water users are positively related to the right to take water. Therefore, in order to gain more water rights and obtain greater economic benefits, certain sub-regions located in the same basin will be motivated to cooperate. However, the previous studies mentioned above did not take into account the cooperation between sub-regions. Some scholars use the concept of cooperation in game theory in multi stakeholder multi reservoir operation system and find that cooperation between reservoirs can create greater economic benefits (Madani and Hooshyar, 2014 [18]; Shen et al., 2018 [19]; Xu et al., 2018 [20]). Similarly, the water distribution system also involves the sharing of water resources by multiple stakeholders (sub-regions). Thus, some studies have focused on the problem of basin water resource allocation from the perspective of cooperative game theory, and many models have been proposed (Guo et al., 2012 [21]; Degefu et al., 2016 [22]). For example, Wang et al developed a basin-wide cooperative water resource allocation model. Sadegh et al developed two new solution concepts for fuzzy cooperative games, namely, Fuzzy Least Core and Fuzzy Weak Least Core, to allocate available water resources and associated benefits to water users in a river basin. The existing research on water resource allocation only uses a single-level planning method. Although some scholars consider the Stackelberg game relationship between basin authority and sub-regional managers when making water resource allocation plans, they use the bi-level optimization model to further narrow the gap with reality. However, the possibility of cooperation among sub-regions is not considered. In recent years, many scholars have begun to study the allocation of water resources in the case of cooperation between sub-regions, but they have not considered the impact of information asymmetry on water distribution. In the actual water resource distribution, the basin authority and the managers of each sub-region often work out the water distribution plan together. In addition, in order to achieve greater economic benefits, cooperation among sub-regions also exists. However, basin authority may be unaware of the cooperation among sub-regions, which will lead to information asymmetry between basin authority and sub-region managers.

Therefore, in this study, we established a bi-level basin water resource optimization model for cooperation and non-cooperation between sub-regions. With respect to alliances among sub-regions, the symmetry of information was considered. The information asymmetry mentioned in this paper refers to the information asymmetry between basin authority and sub-regional managers. Specifically, in order to achieve greater economic benefits, there may be a possibility of cooperation among sub-regions. However, the basin authority does not know that these sub-regions have already achieved cooperation among them, which is so-called cooperation information asymmetry. The main contributions of this paper are as follows:

(1) A bi-level regional water resource allocation model with multi-followers is established, which can better represent the actual watershed distribution model.
(2) This paper considers not only the cooperation among sub-regions, but also the impact of information asymmetry on regional water resources allocation.
(3) The results of this study will help basin authority and sub-regional managers to improve their water diversion plans.

The structure of this paper is as follows: the next section introduces some features of bi-level water resource optimization in the sub-region, including cooperation and non-cooperation; the Section 3 explains the mathematical expression of the model in detail; the Section 4 applies the bi-level planning water resource allocation model to the Qujiang River and presents the results and discussion. Finally, Section 5 offers some concluding remarks and directions for future study.

## 2. Problem Statement 

In this study, we consider the design of optimal allocation of regional water resources and allocation of cooperative benefits when there are multiple sub-regions co-located in a river basin. Each sub-area has one or more reservoirs for storing water, but in order to simplify, we combine the reservoirs in each sub-area into one reservoir. The maximum water storage capacity of this reservoir is the sum of the water storage capacity of all reservoirs in this sub-area. The water source for each sub-area includes water distributed by the basin authority and other sources (including rainfall and groundwater). The model in this study includes two levels of decision makers—the basin authority (leader) and sub-regions (followers). The basin authority, acting on behalf of the public, believes that the public interest is beneficial to the entire local community. Each sub-region pursues its own interests under non-cooperation; in the case of cooperation, members of the alliance pursue the maximization of the overall interests of the alliance, and the remaining sub-regions still pursue the maximization of their own interests. In addition, the annual water distribution plan is considered in this paper, and the symbols involved are based on the year.

**Figure 1:**
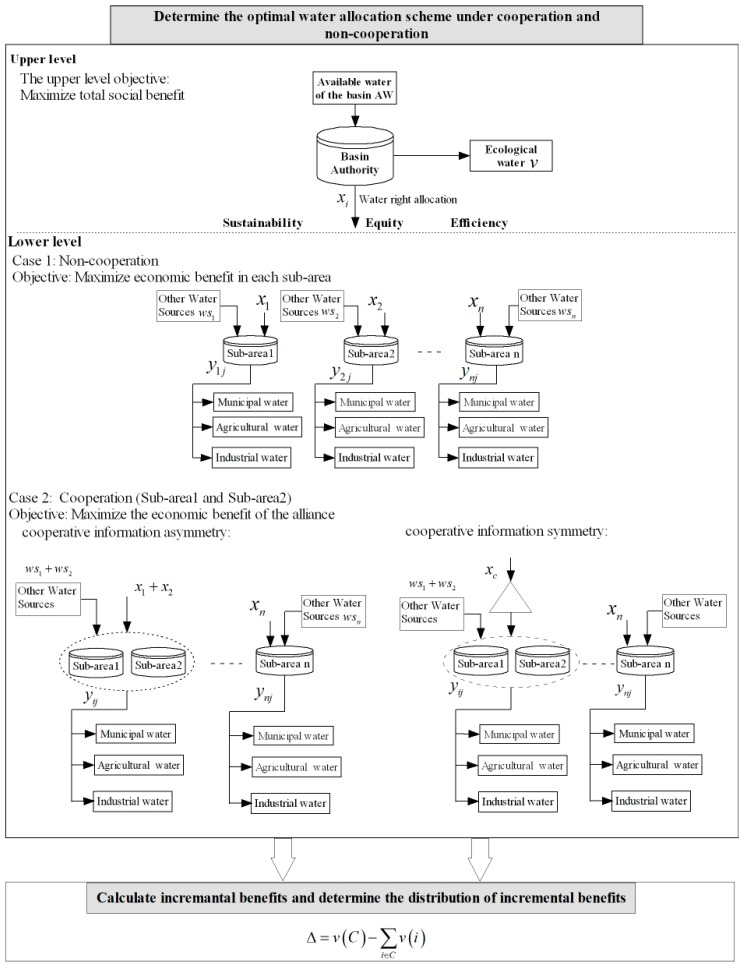
Process for determining the optimal water allocation scheme under cooperation and non-cooperation.

The whole process of the bi-level distribution model includes two steps. First, the basin authority takes action according to the principle of fairness, efficiency, and sustainability. It considers the available water of the basin and allocates water rights according to the society’s ecological environment and for each sub-region to maximize the overall benefits to society. Then, sub-regional managers reallocate the water to water users (i.e., industrial, municipal, and agricultural water users) to achieve the goal of maximizing their own benefits (no-cooperation) or maximizing the overall benefits of the alliance (cooperation). The behavior between the two levels of decision makers in the model is the Stankelberg game. In order to achieve greater economic benefits, there may be a possibility of cooperation among sub-regions. However, the basin authority does not know that these sub-regions have already achieved cooperation among them. Therefore, in the case of cooperation between sub-regions, we consider whether information concerning cooperation is symmetrical, that is, whether the basin authority knows the formation of alliances between sub-regions. When knowledge of cooperation is asymmetrical, the decision of the superior and the sub-area outside the alliance are the same as that of case 1 (non-cooperation; Figure 1), and the sub-areas forming the alliance will integrate the total water that can be allocated to jointly determine the water withdrawal of the users to maximize the economic benefits of the alliance. When cooperative information is symmetrical, the basin authority allocates a total amount of water to the alliance, and the alliance decides how to allocate it, and the basin authority can predict the decision of the sub-region to further optimize its objective function (Figure 1). 

Accrual of an incremental benefit through alliance is the motivation for cooperation among sub-regions. In addition, the equitable allocation of benefits of cooperation between sub-regions is the basic condition for the maintenance of cooperation. The incremental benefit is defined as the total revenue earned by the alliance in excess of total revenue obtained from the sub-regions within the alliance under non-cooperation. Incremental benefit can be characterized as $\Delta =v\left(C\right)-\sum_{i\in C}v\left(i\right)$, where v(C) is the total economic benefit value of the two sub-regions when they cooperate, and $\sum_{i\in C}v\left(i\right)$ is the sum of the economic benefit values when they do not cooperate.

The following section introduces details of the bi-level water resource allocation model of cooperation (information symmetry and asymmetry) and non-cooperation among sub-regions, including the objective function and constraints.

## 3. Modeling

### 3.1. Level of Decision-Making for Basin Authority

Total economic benefits of the society: As an upper-level decision maker, the basin authority represents the public and considers the public interest that benefits the region and society as a whole (Lu et al., 2009 [23]; Tu et al., 2015 [24]). In the bi-level optimization problem, the basin authority takes action first, considering ecological sustainability, formulating a fair and effective distribution plan, and assigning initial water rights to each sub-region to achieve the goal of the greatest overall social benefit F (10^10^ Yuan).

(1) $$\underset{x_{i}}{Max}F=ev+\sum_{i=1}^{n}\sum_{j=1}^{m}p_{j}y_{ij}+\sum_{i=1}^{n}f_{i}\;(\mathrm{non}-\mathrm{cooperation})$$

(2) $$\underset{x_{i}}{Max}F=ev+\sum_{i=1}^{n}\sum_{j=1}^{m}p_{j}y_{ij}+\left[f\left(C\right)+\sum_{i\in N\backslash C}f_{i}\right]\;(\mathrm{cooperation})$$

The objective function consists of three parts: the first part is the ecological benefit ev, where v (10^7^ m^3^)is the public ecological water right to meet the ecological needs of the basin, e (Yuan/m^3^) is the ecological benefit parameter of the basin unite ecological water use; the second part is the payment of the sub-areas’ water costs, where i (i∈N={1,2,⋯,n}) is the index of sub-area in the basin, j (j∈M={1,2,⋯,m}) is the index of water user, $y_{ij}$ (10^7^ m^3^) is the decision variable for the sub-area, $p_{j}$ (Yuan/m^3^) is the unit water cost for user j (Appendix A). For municipal, agricultural and industrial water users, they need to pay water fees to the basin authority for their utilization of water resources, which is the cost of water for the sub-region managers, while for the basin authority, it is the income from their distribution of water resources for the use of water users. The third part is the total economic benefit of the sub-region, $\sum_{i=1}^{n}f_{i}$ indicates the total economic benefit of the region without cooperation, where $f_{i}$ (10^10^ Yuan) is the objective function of sub-region i. In the case of cooperation, the regional total economic benefits include two parts, the benefits of the alliance (i.e., f(C)) and the rest of the non-cooperative areas’ total benefit (i.e., $\sum_{i\in N\backslash C}f_{i}$).

Water supply constraints: The water supply of the river basin is limited, and the total available water is AW(10^7^ m^3^/annual). At the same time, the basin authority should consider the principle of efficiency when allocating water and make full use of available water. Therefore, the total available water is allocated to sub-areas and ecological use as much as possible, where $x_{i}$ (10^7^ m^3^) is the initial water rights of the sub-area i and is the decision-making variable of the superior.

(3) $$\overset{n}{\underset{i=1}{\sum }}x_{i}+v=AW$$

Demand constraints: The basin authority must consider environmental sustainability when developing a water allocation plan. Therefore, public ecological water rights should not be lower than the minimum water demand of the river basin’s ecological environment (i.e., $v_{min}$). The amount of water allocated to each sub-area, excluding the loss of water delivery, and other sources of water in the sub-area, should meet the minimum requirements of that sub-area (i.e., $D_{i}^{min}$). Where $\theta_{i}^{loss}$ represents the water-transfer loss rate:

(4) $$v\geq v_{min}$$

(5) $$\left(1-\theta_{i}^{loss}\right)x_{i}+ws_{i}\geq D_{i}^{min}$$

Storage capacity constraints: Each sub-area has a reservoir for storing water. The sum of the amount of water allocated by the basin authority to each sub-area (excluding water loss) and other water sources cannot exceed the maximum storage capacity of the area (i.e., $S_{i}^{max}$).

(6) $$\left(1-\theta_{i}^{loss}\right)x_{i}+ws_{i}\leq S_{i}^{max}$$

Water allocation constraints: The upper decision variables have a non-negative limit because the initial water rights assigned to each region cannot be negative.

(7) $$x_{i}\geq 0$$

### 3.2. Level of Decision-Making for the Manager of a Sub-Area

Economic Benefits: For the lower level of the bi-level optimization model, each sub-regional manager obtains the initial water rights, and then decides how to allocate water to industrial, municipal, and agricultural users to maximize regional economic benefits. After the lower level makes a decision, it will generate economic benefits $\sum_{j=1}^{m}b_{ij}y_{ij}$ and water cost $\sum_{j=1}^{m}p_{j}y_{ij}$, where $b_{ij}$ (Yuan/m^3^) represents the economic benefit generated by the sub-area i allocation unit water to the user j. Each sub-region pursues its own maximum economic benefit and the lowest cost (non-cooperation). In the case of cooperation, the regions within the alliance pursue the maximum overall benefit and the lowest cost of the alliance based on the initial water right obtained through cooperation, while regions outside the alliance still strive for the maximum benefit and the lowest cost.

(8) $$\underset{y_{ij}}{Max}f_{i}=\sum_{j=1}^{m}b_{ij}y_{ij}-\sum_{j=1}^{m}p_{j}y_{ij}\;(\mathrm{non}-\mathrm{cooperation})$$

(9) $$\underset{y_{ij}}{Max}f\left(C\right)=\sum_{i\in C}\left(\sum_{j=1}^{m}b_{ij}y_{ij}-\sum_{j=1}^{m}p_{j}y_{ij}\right)\;C\subseteq N\;(\mathrm{cooperation})$$

Demand constraints: Each water user has a minimum demand $d_{ij}^{min}$ to meet the most basic needs. In order to balance the development of water users and improve water-use-efficiency, the water volume allocated to the water users by the sub-area should not exceed the normal demand $d_{ij}^{nor}$ of users:

(10) $$d_{ij}^{\min}\leq y_{ij}\leq d_{ij}^{nor}$$

Water supply constraints: The amount of water that can be allocated for each sub-area is limited. Consequently, the sum of the water allocated to each user cannot exceed the distributable water of the area or alliance:

(11) $$\sum_{j=1}^{m}y_{ij}\leq \left(1-\theta_{i}^{loss}\right)x_{i}+ws_{i}\;(\mathrm{non}-\mathrm{cooperation})$$

(12) $$\sum_{i\in C}\sum_{j=1}^{m}y_{ij}\leq \sum_{i\in C}\left(\left(1-\theta_{i}^{loss}\right)x_{i}+ws_{i}\right)\;(\mathrm{cooperation})$$

Water withdrawal constraint: The lower-level decision variables have non-negative restrictions, because the water withdrawal of each user in the sub-region cannot be negative.

(13) $$y_{ij}\geq 0$$

### 3.3. Water Allocation Model under Cooperation and Non-Cooperation

Based on relevant information of sub-areas and available water of the basin, the basin authority determines the initial allocation of water rights to maximize economic benefits to society under the constraints of the existing water supply, demand, storage capacity, and non-negative constraints. The user’s optimal water withdrawal decision is then made in each sub-area to maximize their own economic benefits (non-cooperation) based on the initial water rights of the upper level and other water sources. In the case of cooperation between sub-regions, the sub-regional managers within the alliance, under constraints (10), (12), and (13), jointly make decisions to maximize the overall economic benefits of the alliance (9). Members outside the alliance decide on the optimal water withdrawal right under constraints (10), (11), and (13) to maximize their own economic benefits.

There is an interactive relationship between upper and lower level decision makers in the regional water resource allocation system, which is represented by the Stackelberg game. In order to show the relationship between decision makers, the corresponding objective function and constraints were integrated to obtain the bi-level regional water resource allocation model under non-cooperation (i.e., (14)), cooperative information asymmetry (i.e., (15), and cooperative information symmetry (i.e., (18)):

**Case 1: Non-Cooperation**

(14) $$\begin{array}{l} \underset{x_{i}}{Max}F=ev+\sum_{i=1}^{n}\sum_{j=1}^{m}p_{j}y_{ij}+\sum_{i=1}^{n}f_{i}\\ subject\;\mathrm{to}\{\begin{array}{l} \sum_{i=1}^{n}x_{i}+v=AW\\ v\geq v_{\min}\\ \left(1-\theta_{i}^{loss}\right)x_{i}+ws_{i}\geq D_{i}^{\min}\;\;\forall i\\ \left(1-\theta_{i}^{loss}\right)x_{i}+ws_{i}\leq S_{i}^{\max}\;\;\forall i\\ x_{i}\geq 0\\ \underset{y_{ij}}{Max}f_{i}=\sum_{j=1}^{m}b_{ij}y_{ij}-\sum_{j=1}^{m}p_{j}y_{ij}\\ subject\;\mathrm{to}\{\begin{array}{l} d_{ij}^{\min}\leq y_{ij}\leq d_{ij}^{nor}\;\;\forall i\\ \sum_{j=1}^{m}y_{ij}\leq \left(1-\theta_{i}^{loss}\right)x_{i}+ws_{i}\;\;\forall i\\ y_{ij}\geq 0 \end{array} \end{array} \end{array}$$

Solving this system, the optimal decision and maximum benefit value of basin authority and sub-regions can be obtained as $x_{i}^{*},y_{ij}^{*},F^{*},f_{i}^{*}$.

**Case 2: Cooperation**

Cooperative information asymmetry:

In this case, the basin authority does not know the formation of alliances between sub-regions, and still develops a water allocation plan according to the model without cooperation described above (i.e., (14)). The optimal solutions of the basin authority and sub-areas outside the coalition remain unchanged, and the optimal initial water rights remain $x_{1}^{*}$ for all sub-areas. Suppose sub-regions 1 and 2 now form an alliance. After the basin authority makes the optimal decision, the two sub-regions 1 and 2 of the alliance pursue the maximum overall economic benefits of the alliance, withdrawal of users for the allocated water rights of the alliance is $x_{1}^{*}+x_{2}^{*}$. Sub-regions 1 and 2 obtain the maximum economic benefits of the alliance according to the following model:

(15) $$\begin{array}{l} Maxf_{1,2}=\sum_{i=1}^{2}\left(\sum_{j=1}^{m}b_{ij}y_{ij}-\sum_{j=1}^{m}p_{j}y_{ij}\right)\\ subject\;\mathrm{to}\{\begin{array}{l} d_{ij}^{\min}\leq y_{ij}\leq d_{ij}^{nor}\;\;i=1,2\\ \sum_{i=1}^{2}\sum_{j=1}^{m}y_{ij}\leq \sum_{i=1}^{2}\left(\left(1-\theta_{i}^{loss}\right)x_{i}^{*}+ws_{i}\right)\\ y_{ij}\geq 0\;\;i=1,2 \end{array} \end{array}$$

According to the maximum economic benefit value of sub-areas 1 and 2 obtained by model (14) (i.e., $f_{1}^{*},f_{2}^{*}$) and the maximum economic benefit value of the alliance obtained by model (15) (i.e., $f_{1,2}^{*}$), the incremental benefit under asymmetry of information sharing can be calculated:

(16) $$\Delta =f_{1,2}^{*}-\left(f_{1}^{*}+f_{2}^{*}\right)$$

If the incremental benefit is greater than zero, sub-regions 1 and 2 will have a motivation for cooperation. Taking into account the principle of fairness, sub-regions 1 and 2 are equally distributed with incremental benefits, and the maximum economic benefit value under the asymmetry of cooperative information is obtained:

(17) $$f_{1}^{**}=f_{1}^{*}+\frac{\Delta }{2}\;\;\;\;f_{2}^{**}=\;f_{2}^{*}+\frac{\Delta }{2}$$

Cooperative information symmetry:

In this case, the basin authority is aware of the formation of alliances between certain sub-regions and knows other information, including water demand, and own-sourced water volume. When deciding on the initial allocation of water rights, the basin authority managers, based on the amount of water available for distribution, make decisions to maximize the overall benefits to society, while meeting ecological water requirements, minimum requirements for each sub-region, and maximum water storage constraints. For the sub-areas that form the alliance, the alliance is directly assigned a total amount of water, and the sub-areas within the alliance are no longer allocated separately. Suppose sub-regions 1 and 2 form alliance C, and there is a public reservoir for storing water resources allocated by the basin authority (i.e., $x_{c}$). The maximum water storage capacity of the public reservoir is $S_{c}^{max}$, the rate of water loss is $\theta_{c}^{loss}$, and the minimum demand for alliance C is $D_{c}^{\min}$ ($D_{c}^{\min}=D_{1}^{\min}+D_{2}^{\min}$), $ws_{c}\left(ws_{c}=ws_{1}+ws_{2}\right)$ is the other water source that alliance C can allocate to the water users, then the total amount of water available for the alliance C is $\left(1-\theta_{c}^{loss}\right)x_{c}+ws_{c}$. Sub-areas 1 and 2 are rational subjects, and under the condition of meeting the minimum demand of water users, jointly determine the water withdrawal of the water users to achieve the maximum economic benefit to the alliance. The remaining sub-regions still make decisions based on maximizing their respective economic benefits.

Therefore, the bi-level water resource allocation model under cooperative information symmetry is as follows:

(18) $$\begin{array}{l} \underset{x}{Max\;}F=ev+\sum_{i=1}^{n}\sum_{j=1}^{m}p_{j}y_{ij}+\left[f\left(C\right)+\sum_{i\in N/C}f_{i}\right]\\ subject\;\mathrm{to}\{\begin{array}{c} \begin{array}{l} \sum_{i\in N/C}x_{i}+x_{c}+v=AW\\ v\geq v_{\min}\\ \left(1-\theta_{i}^{loss}\right)x_{i}+ws_{i}\geq D_{i}^{\min}\;i\in N/C\\ \left(1-\theta_{i}^{loss}\right)x_{i}+ws_{i}\leq S_{i}^{\max}\;i\in N/C\\ \left(1-\theta_{c}^{loss}\right)x_{c}+ws_{c}\geq D_{c}^{\min}\\ \left(1-\theta_{c}^{loss}\right)x_{c}+ws_{c}\leq S_{c}^{\max}\\ x_{i},x_{c}\geq 0\\ \underset{y_{ij}}{Max}\;f\left(C\right)=\sum_{i\in C}\left(\sum_{j=1}^{m}b_{ij}y_{ij}-\sum_{j=1}^{m}p_{j}y_{ij}\right)\;i\in C\\ subject\;\mathrm{to}\{\begin{array}{l} d_{ij}^{\min}\leq y_{ij}\leq d_{ij}^{nor}\\ \sum_{i\in C}\sum_{j=1}^{m}y_{ij}\leq \left(1-\theta_{c}^{loss}\right)x_{c}+ws_{c}\\ y_{ij}\geq 0 \end{array}\\ \underset{y_{ij}}{Max}f_{i}=\sum_{j=1}^{m}b_{ij}y_{ij}-\sum_{j=1}^{m}p_{j}y_{ij}\;i\in N/C\\ subject\;\mathrm{to}\{\begin{array}{l} d_{ij}^{\min}\leq y_{ij}\leq d_{ij}^{nor}\\ \sum_{j=1}^{m}y_{ij}\leq \left(1-\theta_{i}^{loss}\right)x_{i}+ws_{i}\\ y_{ij}\geq 0 \end{array} \end{array} \end{array} \end{array}$$

Solving this system, the optimal decision and maximum benefit value of regional authorities and sub-regions under cooperative information symmetry can be obtained: $x_{i}^{**},x_{c}^{**},F^{**},f^{*}\left(C\right),f_{i}^{***}$.

According to the maximum economic benefit value of sub-areas within the alliance under non-cooperation obtained by system (14) (i.e., $\sum_{i\in C}f_{i}^{*}$) and the maximum economic benefit value of the alliance obtained by model (18) (i.e., $f^{*}\left(C\right)$), the incremental benefit under the symmetry of cooperative information can be calculated:

(19) $$\Delta =f^{*}\left(C\right)-\underset{i\in C}{\sum }f_{i}^{*}$$

If the incremental benefit is greater than zero, sub-regions within the alliance will be motivated to cooperate. Taking into account the principle of fairness, they are equally distributed with incremental benefits, and the maximum economic benefit value under the symmetry of cooperative information is therefore:

(20) $$f_{i}^{***}=f_{i}^{*}+\frac{\Delta }{2}\;\left(i\in C\right)$$

## 4. Case Study: Qujiang River Basin

### 4.1. Case Description

Qujiang River Basin flows through three provinces in Southwest China and is the largest tributary of Jialing River. Qujiang River Basin in Sichuan Province supplies water to Bazhong, Nanchong, Guang’an, Guangyuan, and Dazhou sub-area, which has an area of 34,151 km^2^, meeting the water demand of 14.52 million people. Qujiang River Basin supplies 1.998 × 10^10^ m^3^ of water to these five areas every year.

**Figure 2:**
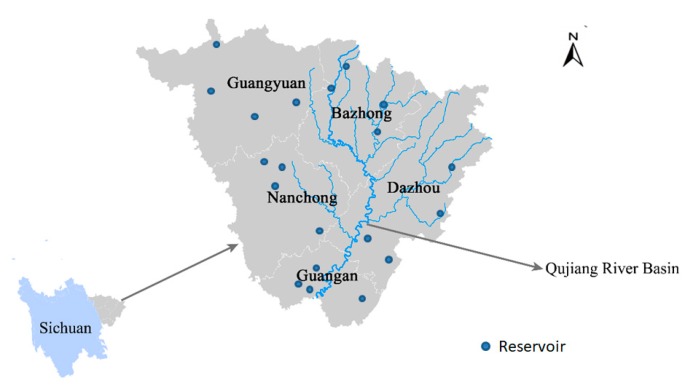
Location of the Qujiang River Basin in Sichuan province.

In recent years, agriculture, industry, and the economy of these sub-regions have developed rapidly, and the demand for water resources has increased significantly, which has aggravated water resource shortages in the Qujiang River Basin. The Qujiang River Basin receives sufficient rainfall during the wet season, and there is a surplus after the needs of the sub-regions are met. However, owing to the uneven spatial and temporal distribution of annual rainfall, the demand for water of these five sub-regions may exceed the available water volume during the dry season. Owing to population growth, industrialization, and urbanization, seasonal water shortages in the Qujiang River Basin are becoming increasingly severe, and the traditional water allocation plans fail to resolve the issues of sustainability and efficiency, resulting in environmental degradation and wasted water. Therefore, to efficiently use the water resources of the Qujiang River Basin to promote the coordinated development of the environment and economy, the basin’s authority and sub-regional managers should respond effectively to these changes. In the Qujiang River Basin, the basin authority and sub-regional managers typically formulate water resource allocation strategies for the following year at the end of the previous. The basin authority allocates water to ecology and the competing sub-regions (Bazhong, Dazhou, Guangan, GuanYuan, and Nanchong), and the sub-regional managers then allocate water to three sectors based on the amount of water stored in the reservoir and distributed by the Qujiang River Basin authority to maximize economic benefits (Figure 2).

Considering the recent rapid economic development of each region, the demand for water resources is increasing, and sub-regional water resource managers hope to obtain more water resources from the basin’s authority to continue to meet the increasing demand of the water users and achieve greater economic benefits. Therefore, there is motivation for cooperation between sub-regional managers. We assume that Bazhong cooperates with Nanchong, as there is a public reservoir in both areas, with a maximum water storage capacity of 1.8 × 10^10^ m^3^ and water loss rate of 0.4. The model presented in this paper is applied to this case to analyze the impacts of sub-regional cooperation and the symmetry of cooperative information on the economic benefits of the basin’s authority and sub-regions. Five sub-areas were considered: Bazhong (BZ, sub-area 1), Nanchong (NC, sub-area 2), Guangan (GA, sub-area 3), GuangYuan (GY, sub-area 4), and Dazhou (DZ, sub-area 5).

Due to the high cost and difficulty in collecting the latest data, the data of this paper mainly comes from the statistical yearbook of Sichuan Province and Sichuan Water Resources Assessment Report, in which the annual available water volume of the basin and the water demand of each water user are calculated based on the data of 2010–2014. The economic benefit value of water users, water storage capacity, water loss rate, and other water source data of each region are from the local sub-region. The lowest annual ecological water requirement in the Qujiang River Basin is 12.3 × 10^7^ m^3^ and the ecological benefit is 25.2 Yuan/m^3^. Detailed data of the water users in the five sub-regions of the Qujiang River Basin are shown in Table 2 and Table 3.

By inputting the data into the proposed model in this study (i.e., systems 14, 15 and 18) and running the solution on Lingo 16.0, we calculated the results in the three modelled cases, which are presented in the following subsection.

### 4.2. Results and Discussion

Scenario 1: The sub-regions did not cooperate. In this case, the managers of the five sub-regions make individual decisions to maximize their respective economic benefits. According to model (14) in this study, the optimal allocation result of water resources without cooperation can be found, as shown in Table 4. It can be seen from Table 4 that when the available water volume is lower than the normal total water demand of the sub-region, the minimum demand of municipal and agricultural users is met, and the water volume of industrial users exceeds the minimum water demand. This is due to the high economic benefit value of industrial water and the pursuit of maximizing economic benefits by basin authority and regional managers. In this case, the overall social benefit value of the Qujiang River Basin was calculated to be 167.938 × 10^10^ Yuan.

Scenario 2: Sub-area 1 and sub-area 2 cooperate and this information is symmetrical. We assumed that the water managers of the two sub-regions BZ and NC reach alliance C, and the Qujiang River Basin Managers know of their cooperation. According to model (18), a set of solutions can be calculated, as shown in Table 5. The initial water rights (676.78 × 10^7^ m^3^) obtained after cooperation between BZ and NC are more than non-cooperation (640.78 × 10^7^ m^3^), and incremental benefits of cooperation were generated (△ = 4.807 × 10^10^ Yuan). In order to share the incremental benefits fairly and maintain the stability of cooperation, the incremental benefits of cooperation were equally distributed between the two regional water managers, and the economic benefits of BZ and NC were 28.107 × 10^10^ Yuan and 20.456 × 10^10^ Yuan, respectively.

From the point of view of water withdraw by water users, the water intake of municipal and agricultural water users in the BZ and NC areas was unchanged. The water intake of industrial water users in the BZ area increased by 77.41% compared with non-cooperation, while industrial users’ water intake in NC just meets their minimum needs. This is because regional water managers pursued the maximum economic benefits on the premise of meeting the minimum needs of municipal and agricultural water users when the water available for distribution does not meet their normal needs.

**Figure 3:**
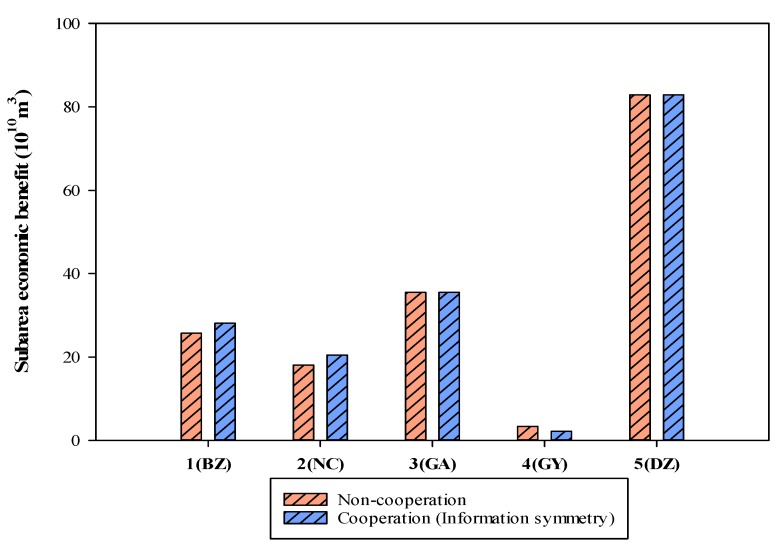
Economic benefit of sub-regions under non-cooperation and cooperation (symmetric information).

Sub-area 1 and 2 form an alliance that does not affect the initial water rights and economic benefits of GA and DZ, while sacrificing the economic benefits of GY. Although the economic benefits of industrial water users in the GY region were second only to DZ, the region was lower than the GA region in terms of average water-use-efficiency. Therefore, the Qujiang River Basin Authority strives to maximize the overall benefits of the basin and sacrifice the benefits of GY. When sub-regions 1 and 2 formed an alliance, the water conflicts in the Qujiang basin were mainly concentrated between alliance C and GY. From the perspective of the Qujiang River Basin administrator, it is clear that the cooperation is conducive to improving its benefits, and the overall social benefit value of the basin increased by 3.699 × 10^10^ Yuan (Figure 3).

Scenario 3: Sub-area 1 and sub-area 2 cooperate and the cooperation information is asymmetric. We assumed that water managers in the two regions BZ and NC have privately reached alliance C. The Qujiang River Basin Authority does not know this. Under this circumstance, the water diversion plan of the Qujiang River Basin Authority remains the same as that of Scenario 1. The GA, GY, and DZ regions’ economic benefits remain unchanged. The water managers in the BZ and NC regions jointly use the total initial water rights. According to model (15), a set of solutions in this case can be calculated (Table 6). A total incremental benefit of cooperation of 0.1 × 10^10^ Yuan was generated. Similarly, the two regions receive equal distributions of these benefits, so the economic benefits of BZ and NC were 25.753 × 10^10^ Yuan and 18.102 × 10^10^ Yuan, respectively. In this case, the overall social benefit value of the Qujiang River Basin was 168.038 × 10^10^ Yuan.

**Figure 4:**
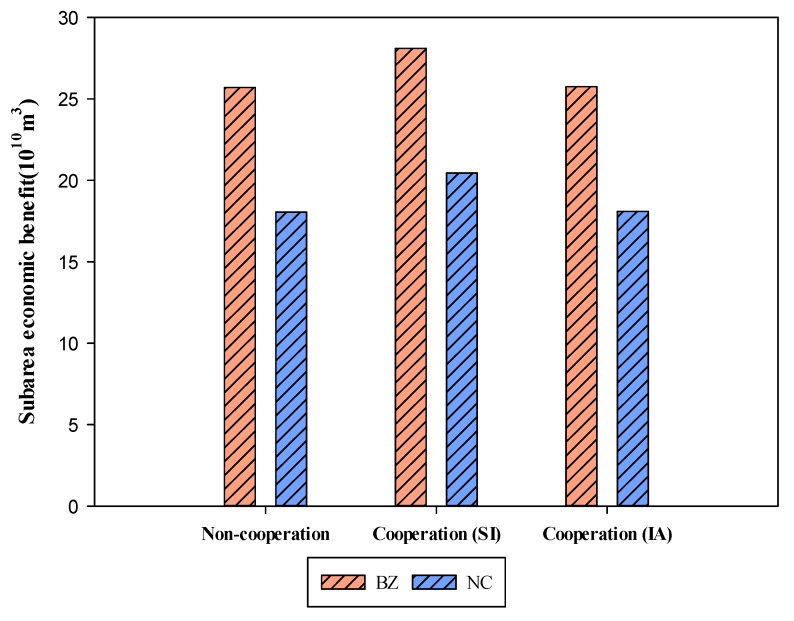
Economic benefits of BZ and NC in different situations. Note: symmetric information (SI), information asymmetry (IA).

In this case, the total resources of the alliance were constant, and the increase in overall interests was at the expense of the less-efficient one (NC)—of industrial water use—and did not affect the interests of the remaining regions and the managers of the Qujiang River Basin. Superficially, this cooperation appears unfavorable to the NC. In fact, this is the redistribution of resources by both partners, which improves the efficiency of resource utilization and achieves greater economic benefits under limited resource conditions. Figure 4 observes that information asymmetry scenario produced improved benefits that are very close to the non-cooperation scenario, but cooperation with symmetry information increased largely the benefits.

From the analysis of the above three different scenarios, some meaningful conclusions and suggestions for the river basin authority can be drawn.

**Proposition** **1.** *Regional cooperation benefited both the cooperative regions and the social welfare value of the entire river basin compared with non-cooperation.*

When the sub-regions cooperated, regardless of whether the basin authority knew of their cooperation, the economic benefit to the cooperating areas and the social welfare value of the entire basin were better than when there was non-cooperation. This is because cooperation between sub-regions has the potential to enable greater economic benefits through efficient allocation of resources. When the sub-regions cooperated and the basin authority did not know about their cooperation, the objective function value increased because of the integration of resources and the redistribution of resources between the cooperating regions. When sub-regional cooperation and the basin authority know of their cooperation, the basin authority prioritizes as much water as possible for the more water-efficient areas. In this case, both the basin authority and the cooperating areas achieve efficient allocation of resources and both sides benefit better than when there was no cooperation.

**Proposition** **2.** *Information symmetry is beneficial to the cooperating regions and the social welfare value of the entire basin compared with information asymmetry.*

When sub-regions cooperate and the cooperation information is symmetrical, the economic benefits of the cooperating areas and the social welfare of the entire basin are better than information asymmetry. When the basin authorities know that some areas of the lower level have formed an alliance, they will sacrifice the benefits of some regions where water use is less efficient and allocate more resources to the alliance that will produce enhanced benefits. Thus, the water resources of the basin have been efficiently distributed as a whole. When information asymmetry, water resources only achieve a partial and efficient allocation in cooperating areas. The overall efficient allocation of resources is better than efficient local allocations.

Therefore, basin authority should encourage cooperation among sub-regional managers, and in the development of watershed water resource allocation plan, the information communication between the upper and lower levels should be strengthened to avoid asymmetric cooperation information. This is because cooperative information symmetry can realize the efficient distribution of water resources in the whole basin, in addition to the efficient utilization of parts of the cooperative regions. In addition, the basin authorities also need to pay attention to the coordinated development of the region and solve the contradiction between the cooperation region and the region with impeded interests. For example, give certain policies and financial support to areas with impaired interests and weak economic development to improve their water-use efficiency. River basin authorities and regional water managers should reduce the rate of water loss as much as possible, thereby reducing water wastage and increasing the amount of water available in each region.

## 5. Conclusions 

In order to study the influence of cooperation among sub-regions and the symmetry of information sharing on the economic benefits of the basin authority and each sub-region, this study proposed a regional water allocation model in three different scenarios. The proposed bi-level optimization model considered the ecological sustainability of the basin, and clearly reflects a Stackelberg game relationship between the basin authority and sub-regional managers. Finally, the model of this study was applied to the Qujiang River Basin, and the decisions of the Qujiang River Basin authority and sub-regional managers were discussed in the three scenarios of non-cooperation, cooperation, where information sharing was symmetrical, or asymmetric. The results demonstrated that cooperation between sub-regions was beneficial to the basin authority and to the cooperative regions compared with non-cooperation; compared with information asymmetry, information symmetry was beneficial to the cooperating areas and the social welfare value of the entire basin. In addition, the asymmetry of information did not affect the interests of the remaining sub-regions.

In the future, the following research directions have potential value. First, although this study considered the three principles of efficiency, fairness, and sustainability, it only considered the single goal of maximizing economic benefits. In future research, we will introduce multi-objective bi-level programming to study the impact of cooperation among sub-regions and symmetry of cooperation information on decisions of leaders and lower levels. Second, examining the impacts of cooperation of three or more sub-regions is also an interesting research direction. Finally, because water allocation is a complex system involving many uncertainties, future research will introduce fuzzy random variables and nonlinear constraints or objective functions.

## Figures and Tables

**Table 1:** Literature Review on Water Allocation.

Relevant Literature	Bi-Level	Cooperation	Information Asymmetry
Xu et al., 2013 [6]	X	-	-
Xu and co-workers, 2017 [12]	X	-	-
Hu and co-workers, 2016 [13]	X	-	-
Madani, 2010 [14]	-	X	-
Wang et al., 2008 [15]	-	X	-
Sadegh et al., 2011 [16]	-	X	-
He et al., 2018 [17]	-	X	-
This paper	X	X	X

**Table 2:** Model Parameters—storage capacity, loss rate, and economic benefit of sub-areas.

Sub-Area i	$\theta_{i}^{loss}$	$ws_{i}\;(10^{7}\;m^{3})$	$S_{i}^{max}\;(10^{7}\;m^{3})$	$b_{ij}\;(\mathrm{Yuan}/m^{3})$		
				Industry	Agriculture	Domestic
1 (BZ)	0.45	428	1000	60.61	42.55	43.86
2 (NC)	0.38	239	800	54.35	34.48	32.26
3 (GA)	0.30	340	800	67.57	47.62	47.17
4 (GY)	0.50	29	600	74.07	31.25	37.88
5 (DZ)	0.32	635	1200	86.96	43.11	45.45

Note: i is a sub-area; $\theta_{i}^{loss}$ is the loss rate; $ws_{i}$ is the other water sources; $S_{i}^{max}$ is the maximize storage capacity, and $b_{ij}$ is the economic benefit.

**Table 3:** Model parameters—requirement of water volumes and unit water costs.

Sub-Area i	Industry			Agriculture			Domestic		
	$d_{i1}^{min}$	$d_{i1}^{nor}$	$p_{1}$	$d_{i2}^{min}$	$d_{i2}^{nor}$	$p_{2}$	$d_{i3}^{min}$	$d_{i3}^{nor}$	$p_{3}$
1 (BZ)	87	488	1.2	319	765	0.2	114	155	1.0
2 (NC)	56	72	1.2	363	420	0.2	57	78	1.0
3 (GA)	164	251	1.2	332	443	0.2	67	96	1.0
4 (GY)	10	28	1.2	34	42	0.2	7	8	1.0
5 (DZ)	324	683	1.2	342	611	0.2	217	281	1.0

Note: $d_{ij}^{min}$ is the minimum water requirement (10^7^ m^3^); $d_{ij}^{nor}$ is the normal water requirement (10^7^ m^3^); and $p_{j}$ is the unit water cost (RMB/m^3^).

**Table 4:** Water allocation result under non-cooperation.

Sub-Area i	Allocation Amount (10^7^ m^3^/Annual)				Economic Benefit (10^10^ Yuan)				
	$x_{i}$	$y_{i1}$	$y_{i2}$	$y_{i3}$	$f_{i}$	$EB_{i1}$	$EB_{i2}$	$EB_{i3}$	$F^{*}$
1(BZ)	232.72	123.00	319.00	114.00	25.703	7.307	13.510	4.886	167.938
2(NC)	408.06	72.00	363.00	57.00	18.052	3.827	12.444	1.782	
3(GA)	442.86	251.00	332.00	67.00	35.496	16.659	15.743	3.093	
4(GY)	80.00	28.00	34.00	7.00	3.354	2.040	1.056	0.258	
5(DZ)	822.06	683.00	342.00	217.00	82.895	58.574	14.675	9.646	

**Table 5:** Water allocation result under cooperation (information symmetry, BZ and NC).

Sub-Area i	Allocation Amount (10^7^ m^3^/Annual)				Economic Benefit (10^10^ Yuan)				
	$x_{i}$	$y_{i1}$	$y_{i2}$	$y_{i3}$	$f\left(C\right)/f_{i}$	$EB_{i1}$	$EB_{i2}$	$EB_{i3}$	$F^{**}$
1(BZ)	676.78	218.21	319.00	114.00	48.562	12.964	13.510	4.886	171.637
2(NC)		56.00	363.00	57.00		2.976	12.444	1.782	
3(GA)	442.86	251.00	332.00	67.00	35.496	16.659	15.743	3.093	
4(GY)	44.00	11.76	34.00	7.00	2.171	0.857	1.056	0.258	
5(DZ)	822.06	683.00	342.00	217.00	82.895	58.574	14.675	9.646	

**Table 6:** Water allocation result under cooperation (information asymmetry, BZ and NC).

Sub-Area i	Allocation Amount (10^7^ m^3^/Annual)				Economic Benefit (10^10^ Yuan)				
	$x_{i}$	$y_{i1}$	$y_{i2}$	$y_{i3}$	$f_{1,2}$	$EB_{i1}$	$EB_{i2}$	$EB_{i3}$	$F^{**}$
1(BZ)	640.78	138.99	319.00	114.00	43.855	8.257	13.510	4.886	168.038
2(NC)		56.00	363.00	57.00		2.976	12.444	1.782	

## Appendix A. Nomenclatures

1. **Subscripts:**
  i = index of sub-area in the river basin, where, i=1,2⋯n;
  j = index of water user in the sub-area, where j=1,2⋯n;
2. **Decision Variables:**
  v = public ecological water rights to maintain the ecological environment of the river basin (10^7^m^3^/Annual);
  $x_{i}$ = initial water rights of sub-area i, determined by the upper level decision maker (10^7^m^3^/Annual);
  $y_{ij}$ = water withdrawal of user j in sub-area i, determined by the lower level decision maker (10^7^m^3^/Annual);
3. **Objective function:**
  Max F = Objective function of Basin Authority (Maximize total social benefit, 10^10^ Yuan);
  Max $f_{i}$ = Objective function of sub-area i (Maximize economic benefit in each sub-area, 10^10^ Yuan);
4. **Parameters:**
  AW = available water in the river basin (10^7^m^3^/Annual);
  $v_{min}$ = minimal ecological water requirement of the river basin (10^7^m^3^/Annual);
  e = the ecology efficiency parameter for the river basin in unit of water supply (Yuan/m^3^);
  $p_{j}$ = the unit water cost for user j (Yuan/m^3^);
  $S_{i}^{max}$ = maximum storage capacity of sub-area i (10^7^m^3^/Annual);
  $\theta_{i}^{loss}$ = water transfer loss ratio from basin authority to sub-area i;
  $ws_{i}$ = other water sources such as the rainfall and the groundwater in sub-area i (10^7^m^3^/Annual);
  $b_{ij}$ = benefit parameter for water user j in sub-area i per unit of water allocated (Yuan/m^3^);
  $d_{ij}^{min}$ = minimal water requirement for user j in sub-area i (10^7^m^3^/Annual);
  $d_{ij}^{nor}$ = normal water requirement for user j in sub-area i (10^7^m^3^/Annual);
  $EB_{ij}$ = Total economic benefit value of water user j in sub-area i (10^10^ Yuan/Annual).
